# Association between Allergic Rhinitis and Regular Physical Activity in Adults: A Nationwide Cross-Sectional Study

**DOI:** 10.3390/ijerph17165662

**Published:** 2020-08-05

**Authors:** Jewel Park, Joo Hee Park, Jaehyung Park, Jimi Choi, Tae Hoon Kim

**Affiliations:** 1 Korea University College of Medicine, Seoul 02841, Korea; bakjewel@korea.ac.kr (J.P.); yourstrulyjoohee@korea.ac.kr (J.H.P.); godz131@korea.ac.kr (J.P.); 2 Department of Biostatistics, Korea University College of Medicine, Seoul 02841, Korea; camellia11@korea.ac.kr; 3 Department of Otorhinolaryngology-Head and Neck Surgery, Korea University College of Medicine, Seoul 02841, Korea

**Keywords:** allergic rhinitis, rhinoscopy, IgE, physical activity, ARIA

## Abstract

Evidence regarding the association between allergic rhinitis (AR) and physical activity (PA) is conflicting. Previous studies have mostly relied only on self-reported symptoms to define AR, did not classify AR by severity or persistence, and included only children or athletes. The present cross-sectional study evaluated the association between PA and objectively-defined AR and its subtypes in the general adult population using data for 1932 eligible participants aged 19 years or older in the 2010 Korea National Health and Nutrition Examination Survey. Multivariable logistic regression analyses were performed to evaluate the relationship between three types of PA and overall AR, AR subtypes, and rhinoscopy findings showed that moderate-severe AR was positively associated with vigorous (odds ratio [OR] = 3.392, *p* = 0.002) and moderate (OR = 3.623, *p* = 0.007) PA compared to mild AR, while persistent AR was associated with vigorous (OR = 3.954, *p* = 0.004) and moderate (OR = 3.411, *p* = 0.022) PA compared to intermittent AR. On rhinoscopy, vigorous PA was significantly associated with watery rhinorrhea (OR = 2.203, *p* = 0.048) but not pale mucosa. Total immunoglobulin E (IgE) and three allergen-specific IgE were not significantly elevated in participants who performed PA. Therefore, regular vigorous PA is associated with subjective and objective aggravation of AR symptoms, which may not necessarily manifest as increased serum IgE levels.

## 1. Introduction

Allergic rhinitis (AR) is one of the most common health conditions worldwide and is estimated to affect more than 500 million people; while the prevalence varies based on age and region, the prevalence is rising in most countries [1]. The symptoms of AR include itching, nasal congestion, sneezing, and rhinorrhea [2], which may significantly impair the quality of life, sleep, cognitive function, and work productivity [3] as well as the social life and emotional health of patients [4]. The well-established risk factors of AR include family history, environmental pollution, and exposure to allergens; however, whether physical activity (PA) is also a risk factor is unclear.

PA is associated with a wide range of health benefits and is strongly recommended to improve health; however, in some instances, it can elicit dysregulated inflammatory responses and exacerbate pre-existing illnesses such as asthma, cystic fibrosis, and systemic lupus erythematosus [5]. Regarding AR, studies conducted between the 1960s and 1990s [6,7,8] showed that PA decreases nasal resistance, thereby improving AR symptoms. However, these studies mainly observed an acute change in symptoms during PA, but not the long-term effects. These studies also did not consider other symptoms of AR such as rhinorrhea, which is more frequently reported in AR patients performing PA [9]. Thus, the association between PA and AR remains controversial, with some cross-sectional studies showing an increased risk of AR symptoms in those performing vigorous PA [10,11] and others reporting decreased risk [12,13,14]. The high prevalence of AR in athletes who perform vigorous PA suggests that the PA intensity may influence the association between PA and AR [15].

Almost all previous studies on the association between AR and PA have used self-reported symptoms of rhinitis to define AR. However, this definition may include non-allergic rhinitis (NAR) or occupational rhinitis. Thus, allergen-specific immunoglobulin E (IgE) reactivity must be present to confirm a diagnosis of AR and clinical examination may improve the accuracy of the diagnosis [16]. These studies have also viewed AR as a single disease; however, AR can be classified by severity and persistence and the risk factors and allergen sensitization patterns may differ according to this classification [14,17,18]. Finally, most studies have assessed the above associations in children; however, the characteristics of AR may differ significantly between children and adults [19]. Therefore, the present study analyzed the association between objectively-measured AR and three intensities of PA (vigorous PA, moderate PA, walking) in adults using data from the Korea National Health and Nutrition Examination Survey (KNHANES).

## 2. Materials and Methods

### 2.1. Survey Used for Data Collection and the Study Population

The KNHANES is a nationwide, population-based, cross-sectional health examination and survey conducted to understand the health and nutrition status of the Korean population since 1998. The KNHANES is conducted annually by randomly sampling 23 households in each of the 192 regions and includes 10,000 household members over one year of age. Foreigners, people enlisted in the army, people in jail, and people in nursing homes are excluded from the survey. The survey consists of a health interview (e.g., smoking status, physical activity, use of medical services), nutritional survey (e.g., frequency and amount of food items consumed, nutritional supplements), and clinical examinations (e.g., blood tests, urine analysis, otorhinolaryngology examination).

**Figure 1:**
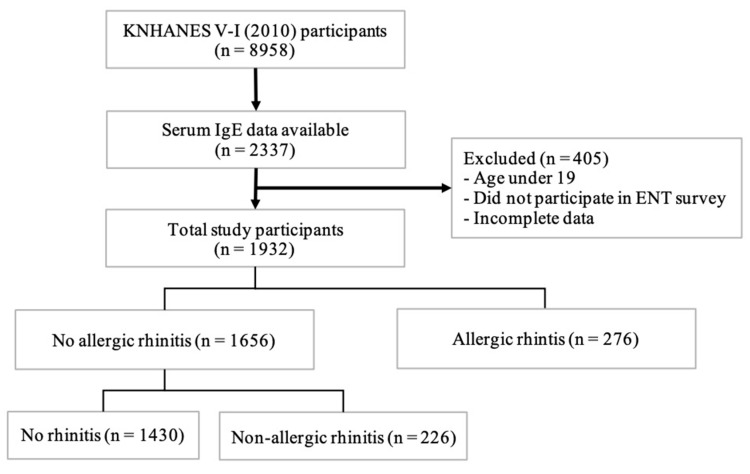
Flowchart used for participant selection. KNHANES, Korea Health and Nutrition Examination Survey; IgE, immunoglobulin E; ENT, Ear, Nose, and Throat.

The present study used data collected by the KNHANES V-1 conducted in 2010, which was the only year in which data for serum IgE levels were collected. In particular, an otorhinolaryngology survey and endoscopic examinations were completed by 150 residents from the otorhinolaryngology departments of 47 institutes. Before participating in the survey, the residents were educated on the following: the overview of the survey; purpose of, and caution related to, the investigation of otolaryngology diseases; detailed guidance on the diagnosis and severity classification of diseases; and simulation training with the actual examination equipment. Among the 8313 subjects who participated in the otorhinolaryngology survey, 1932 were 19 years of age or older and underwent IgE testing, and thus, were included in this study (Figure 1). All participants were informed of the purpose and use of the data collected and provided written consent before beginning the survey sessions. The study was approved by the Institutional Review Board of the Korea Centers for Disease Control and Prevention (2010-02CON-21-C).

### 2.2. Assessment of Variables

AR was defined as having replied “yes” to the question “In the past year, have you experienced rhinitis symptoms such as sneezing, rhinorrhea, nasal obstruction, and nasal itching that were unrelated to the common cold (fever, sore throat)?” as well as positivity for at least one allergen-specific IgE (>0.35 kU/L). NAR was defined as having replied “yes” to the above question but being negative for any of the specific IgE. AR was further classified according to the Allergic Rhinitis and its Impact on Asthma (ARIA) guidelines based on severity (mild/moderate-severe) or persistence (intermittent/persistent) [1]. Rhinoscopy was used in clinical examinations to determine whether the participants had rhinorrhea or pale nasal mucosa. Total IgE and three allergen-specific IgE (*Dermatophagoides farinae*, dog, cockroach) were measured using ImmunCAP 100 (Phadia, Uppsala, Sweden) and were logarithmically transformed (base 10) for statistical analyses [20].

In the KNHANES V, the participants are asked three questions regarding PA: (1) “In the past week, on how many days have you performed more than 10 min of vigorous physical activity that made you extremely tired or more out of breath than usual (e.g., work-related or athletic activity such as running (jogging), hiking, cycling at high speeds, fast swimming, soccer, basketball, jump rope, squash, singles tennis, lifting heavy weights, etc.)?”; (2) “In the past week, on how many days have you performed more than 10 min of moderate physical activity that made you extremely tired or more out of breath than usual (e.g., work-related or athletic activity such as slow swimming, doubles tennis, volleyball, badminton, lifting light weights, etc., except walking)?”; and (3) “In the past week, on how many days have you walked more than 10 min?” If subjects performed any of the three PAs on even a single day, they were also asked for how many minutes they performed the activity per day, on average. Then, we determined whether the participants had satisfied the recommendations of the American College of Sports Medicine for each PA: minimum 20 min of vigorous PA a day, at least three times a week; minimum 30 min of moderate PA a day, at least five times a week; or minimum 30 min of walking a day, at least five times a week [21].

The health interview collected information on educational level, marital status, household number, household income, occupation type, residential location, smoking habits, alcohol consumption, body mass index (BMI), sleep hours, stress level, asthma, and atopic dermatitis. Participants who had graduated high school and above were categorized as having a high educational level. Households with income in the upper two quartiles were categorized as having a high household income. Current smokers were defined as those who had smoked at least five packs of cigarettes in a lifetime and who reported smoking “every day” or “frequently.” Alcohol consumption was determined by asking the participants whether they consumed alcohol at least once per month in the past year. Participants with a BMI <25 and ≥25 kg/m^2^ were categorized as normal and obese, respectively. In response to the statement “Usual perception of stress”, participants who replied “extremely stressed” or “quite stressed” were categorized as high-stress, while those who replied “slightly stressed” or “rarely stressed” were categorized as low-stress. Sleep hours were divided into ≥8 and <8 h. Participants who replied “yes” to the statement “I have experienced asthma” were categorized as having asthma and those who replied “yes” to the statement “I have experienced atopic dermatitis” were categorized as having atopic dermatitis.

### 2.3. Statistical Analysis

Complex sample analysis was performed using the stratification, cluster, and sample weight variables provided in the KNHANES dataset. Weighted numbers and percentages were calculated by multiplying the sample weight variable by unweighted sample values. For the differences between the baseline characteristics of AR and control groups, continuous variables were analyzed using Student’s *t*-test for weighted means, while categorical variables were analyzed using Rao–Scott chi-square tests. Logistic regression analysis was performed to calculate the odds ratios (ORs) between PA and overall AR in the total study population and the associations between PA and AR subtypes or rhinoscopy findings in the AR population. Multiple logistic regression model was used to adjust for confounding factors including age, sex, and other variables with *p*-values less than 0.2 in Table 1 (residence, household number, education, marriage, asthma, atopic dermatitis, stress). *p*-values less than 0.05 were considered statistically significant. All statistical analyses were performed using IBM SPSS Statistics for Windows, version 24.0 (IBM Corp., Armonk, NY, USA), SAS software, version 9.4 (SAS Institute Inc., Cary, NC, USA), or R software, version 3.6.0 (R foundation for Statistical Computing, Vienna, Austria)

## 3. Results

### 3.1. Baseline Characteristics

Among the 1932 participants, 276 (15.1%) belonged to the AR group (Table 1). The AR group was younger than the control group (39.68 ± 1.30 vs. 45.64 ± 0.59) (*p* < 0.001). Compared to the control group, the participants in the overall AR group were more likely to be unmarried, have graduated high school, and have a higher level of stress. However, they did not differ in sex, location of residence, household income, household number, and occupation. Additionally, the overall AR group reported a higher prevalence of asthma and atopic dermatitis but not alcohol use, smoking, obesity, and sleep.

### 3.2. Association between AR and PA in the Total Study Population

The association between overall AR and the three PA intensities was analyzed in the total study population (Table 2). The proportions of the overall AR group who performed vigorous PA (18.2% vs. 17.1%) and walking (44.8% vs. 40.6%) were slightly higher than those of the control group; however, the OR in univariate logistic regression was not significant for all three intensities of PA (i.e., vigorous PA (OR = 1.080, *p* = 0.696), moderate PA (OR = 0.914, *p* = 0.714), and walking (OR = 1.185, *p* = 0.316) (Table 2)).

### 3.3. Associations between AR and PA in the AR Population

The study population was limited to 276 participants of the AR group, of whom 91 (33.1%) reported having moderate-severe AR and 78 (26.8%) reported having persistent AR (Table 3). Univariate logistic regression showed that moderate-severe AR and persistent AR were both significantly associated with vigorous PA and moderate PA. After adjusting for confounding factors, moderate-severe AR showed positive correlations with vigorous (OR = 3.392, *p* = 0.002) and moderate (OR = 3.623, *p* = 0.007) PA, as did persistent AR with vigorous (OR = 3.954, *p* = 0.004) and moderate (OR = 3.411, *p* = 0.022) PA. In comparison, participants who had NAR (i.e., those with allergen-specific IgE levels <0.35 kU/L for all three allergens) did not show positive correlations between the severity or persistence of rhinitis symptoms and PA performance (Supplementary Table S1).

These significant associations between PA and self-reported symptoms of AR were further explored by analyzing the association between PA and rhinoscopy findings (Table 4) or serum IgE levels (Table 5). After adjusting for confounding factors, watery rhinorrhea was positively correlated with vigorous PA (OR = 2.203, *p* = 0.048). While pale mucosa was more commonly found in participants performing vigorous or moderate PA, these associations were not significant in univariate or multivariate logistic regression. Total IgE and the three allergen-specific IgE levels were not significantly associated with any of the three types of PA. Differences in allergen-specific IgE levels (divided into six grades) between participants who performed PA and those who did not are shown in Supplementary Table S2.

## 4. Discussion

The main finding of this study was that increased AR severity or persistence was related to higher rates of PA performance in adults. To our knowledge, this is the first detailed analysis of the association between AR and PA to classify AR by severity or persistence and PA by intensity. By diagnosing AR not only from clinical symptoms but also using allergen-specific IgE levels and by supplementing this diagnosis with rhinoscopy findings, we improved the reliability and the robustness of our results and reduced the risk of including non-AR patients, who accounted for approximately 45% of the participants complaining of rhinitis symptoms in our survey. By using a cross-sectional survey, our study is representative of the general adult population, unlike previous studies that have mostly focused on children or athletes.

While no apparent association was found between overall AR and PA, classifying AR according to the ARIA (Allergic Rhinitis and its Impact on Asthma) guidelines revealed significant associations. In our study, AR patients who performed vigorous PA were more likely to have more severe or persistent symptoms and watery rhinorrhea on rhinoscopy. A previous study in children also showed that the association between AR and PA differed significantly after dividing patients by symptom severity, suggesting that subgroup analyses of AR patients can provide useful insights [19]. Furthermore, the fact that significant associations were found for vigorous and moderate PA, but not for walking, highlights the importance of analyzing different types of PA [22].

The pathophysiology behind exercise-induced AR remains unclear. One possible hypothesis is the T helper 2 (Th2) allergic response, which involves the release of cytokines such as interleukin (IL)-4, IL-5, and IL-13 [16]. This shift of Th1/Th2 balance toward the Th2 response also occurs after strenuous exercise, in which the expression of Th2-associated genes is upregulated [23,24]. A study in rats also showed that excessive exercise over nine weeks of training resulted in a shift toward the Th2 response, with higher levels of IL-4 and lower levels of interferon (IFN)-γ post-training [24]. However, our study showed that AR patients who performed PA did not have higher levels of allergen-specific IgE, which plays a major role in the early phase of the Th2 response by activating mast cells. This may be because, although IgE levels increase post-exercise, they return to baseline levels as early as 1 h after exercise [25], while the symptoms can persist independent of IgE through a late-phase reaction [16]. Future studies measuring Th2 cytokines in AR patients after PA may help to elucidate the role of the Th2 response in exercise-induced AR.

In addition to the allergen-specific IgE data, the rhinoscopy findings add to the robustness of our results. We observed that vigorous PA was positively correlated with watery rhinorrhea, but not pale mucosa. Vigorous PA and the resulting inhalation of cold and dry air may stimulate cholinergic receptors in the airways and the enhanced cholinergic glandular secretory activity may be responsible for eliciting rhinorrhea [9]. While this response is not allergic in nature, the late-phase reaction of the above-mentioned Th2 response may render the nasal mucosa hyper-reactive and may explain the watery rhinorrhea in these patients. While pale mucosa, another characteristic rhinoscopy finding in AR, was not significantly increased in patients performing PA, this characteristic may not have been present at the time of inspection because it occurs in the relatively short early-phase response [26].

Another possible mechanism is the exercise-induced neutrophilic infiltration of the nasal mucosa. Athletes may have an increased number of neutrophils in their nasal mucosa after exercise, which is accompanied by increased AR symptoms, especially rhinorrhea [27,28]. In vitro experiments suggest that activated neutrophils can prime T cells and attract eosinophils, thereby contributing to allergic inflammation in AR patients [29].

It is worth comparing the association between AR symptoms and PA with exercised-induced bronchoconstriction (EIB), which occurs in approximately 90% of asthmatic individuals [30]. AR is strongly associated with asthma, with striking pathophysiological similarities, and almost all adults with asthma also suffer from rhinitis [31]. Importantly, exercise is not a risk factor for asthma, but rather a trigger for the aggravation of symptoms in patients who already have asthma [32]. Our result is an interesting parallel, as PA was not significantly associated with overall AR, but was associated with increased rhinitis symptom severity and persistence in patients with AR. The exact pathophysiology of EIB in asthmatic patients is not clear; however, cold and dry air is thought to aggravate symptoms by stimulating the release of inflammatory mediators [33]. Whether exercise can exacerbate AR symptoms through similar mechanisms or otherwise remains to be elucidated.

The present study has several limitations. First, based on the cross-sectional study design, the causal relationship between PA and AR is difficult to determine due to the simultaneous collection of information from the survey. Second, because the KNHANES data only included sensitization to three indoor allergens (*Dermatophagoides farinae*, dog, and cockroach), it could not capture AR patients who were sensitized to other allergens. In particular, pollen is an important outdoor allergen that could cause AR symptoms in participants who perform PA outdoors; however, specific IgE against pollen was not measured in the KNHANES. Third, it was not possible to differentiate whether patients performed PA indoors or outdoors; thus, it is not clear whether the AR symptoms were dependent on exposure to specific allergens or the intensity of PA. Finally, the data used in this study were collected in 2010; therefore, the context and relationship between analyzed parameters may have changed since then. Despite these limitations, the strength of this study is that objective and robust data were used to diagnose and analyze AR and that classifying AR and PA provided a deeper understanding of the associations.

## 5. Conclusions

In this nationwide study, AR was associated with PA depending on the classification of AR and type of PA (i.e., regular performance of vigorous PA was associated with subjective and objective aggravation of AR symptoms). While further studies are needed to validate this relationship, clinicians and AR patients may consider vigorous PA as a potential risk factor for the aggravation of AR symptoms and modify the patients’ behavior to avoid aggravating symptoms such as performing less vigorous PA or improving adherence to environmental exposure to allergens.

## Figures and Tables

**Table 1:** Baseline characteristics of adults with and without allergic rhinitis according to KNHANES 2010.

	AR (−)	AR (+)	*p*
	n = 1656, N = 31,667,729	n = 276, N = 5,615,440	
	84.90%	15.10%	
Age	45.64 ± 0.59	39.68 ± 1.3	<0.001
Sex (female)	851 (51.1)	130 (46.2)	0.238
Residence (urban)	1314 (76.9)	231 (82.3)	0.113
Household income (Upper 50%)	930 (53.7)	158 (55.0)	0.758
Household number (≥4)	732 (48.0)	134 (53.4)	0.179
Marriage status (married)	1354 (80.3)	178 (63.9)	<0.001
Education (≥12 years)	1141 (68.2)	225 (77.8)	0.016
Occupation (white collar)	1193 (71.7)	211 (74.8)	0.341
Alcohol use (Yes)	960 (59.1)	162 (55.8)	0.362
Smoking (Yes)	416 (27.5)	76 (29.0)	0.685
Obesity (BMI ≥ 25)	542 (34.0)	73 (29.7)	0.257
Sleep (≥8 h)	480 (29.8)	88 (30.4)	0.870
Stress (High)	430 (27.4)	87 (35.7)	0.034
Asthma	53 (3.4)	23 (9.4)	0.001
Atopic dermatitis	83 (5.3)	26 (9.9)	0.010

n = unweighted number of study population, N = weighted number of population. KNHANES, Korea National Health and Nutrition Examination Survey.

**Table 2:** Odds ratios for the association between physical activity and prevalence of allergic rhinitis in the total study population.

	n (%)		Univariate		Multivariate	
	AR (−)	AR (+)	Odds Ratio (95% CI)	*p*	Odds Ratio (95% CI)	*p*
	n = 1656	n = 276				
Vigorous	272 (17.1)	52 (18.2)	1.080 (0.731–1.595)	0.696	0.965 (0.655–1.423)	0.857
Moderate	177 (12.0)	33 (11.1)	0.914 (0.565–1.480)	0.714	0.998 (0.617–1.614)	0.993
Walking	681 (40.6)	117 (44.8)	1.185 (0.849–1.654)	0.316	1.126 (0.804–1.577)	0.888

n = unweighted number of study population. AR, allergic rhinitis. Multivariate analysis was adjusted for age, sex, residence, household number, education, marriage, asthma, atopic dermatitis, and stress.

**Table 3:** Odds ratios for the associations between physical activity and rhinitis (A) severity or (B) persistence in patients with allergic rhinitis.

**A**						
	**n (%)**		**Univariate**		**Multivariate**	
	**Mild**	**Moderate–Severe**	**Odds Ratio** **(95% CI)**	***p***	**Odds Ratio** **(95% CI)**	***p***
	**n = 183**	**n = 91**				
Vigorous	30 (11.8)	22 (31.2)	3.403 (1.597–7.249)	0.002	3.392 (1.555–7.398)	0.002
Moderate	18 (6.2)	15 (21.0)	4.050 (1.667–9.839)	0.002	3.623 (1.444–9.090)	0.007
Walking	73 (42.3	44 (49.8)	1.356 (0.736–2.499)	0.326	1.053 (0.548–2.026)	0.876
**B**						
	**n (%)**		**Univariate**		**Multivariate**	
	**Intermittent**	**Persistent**	**Odds Ratio** **(95% CI)**	***p***	**Odds Ratio** **(95% CI)**	***p***
	**n = 196**	**n = 78**				
Vigorous	30 (13.6)	22 (30.9)	2.848 (1.241–6.539)	0.014	3.954 (1.551–10.084)	0.004
Moderate	20 (7.2)	13 (21.7)	3.555 (1.339–9.440)	0.011	3.411 (1.194–9.748)	0.022
Walking	82 (42.9)	35 (49.9)	1.327 (0.723–2.436)	0.358	1.383 (0.732–2.611)	0.314

n = unweighted number of study population. Multivariate analysis was adjusted for age, sex, residence, household number, education, marriage, asthma, atopic dermatitis, and stress.

**Table 4:** Odds ratios for the association between physical activity and (A) watery rhinorrhea or (B) pale mucosa on rhinoscopy in patients with allergic rhinitis.

**A**						
	**n (%)**		**Univariate**		**Multivariate**	
	**Healthy**	**Watery Rhinorrhea**	**Odds Ratio** **(95% CI)**	***p***	**Odds Ratio** **(95% CI)**	***p***
	**n = 179**	**n = 92**				
Vigorous	52 (14.4)	23 (28.4)	2.351 (1.071–5.159)	0.033	2.203 (1.006–4.825)	0.048
Moderate	23 (10.4)	10 (13.7)	1.378 (0.475–3.996)	0.553	1.199 (0.395–3.641)	0.747
Walking	80 (47.3)	36 (39.0)	0.715 (0.389–1.312)	0.276	0.558 (0.266–1.170)	0.121
**B**						
	**n (%)**		**Univariate**		**Multivariate**	
	**Healthy**	**Pale Mucosa**	**Odds Ratio** **(95% CI)**	***p***	**Odds Ratio** **(95% CI)**	***p***
	**n = 192**	**n = 79**				
Vigorous	36 (17.6)	16 (21.4)	1.279 (0.553–2.955)	0.562	1.293 (0.546–3.063)	0.556
Moderate	25 (10.3)	8 (14.2)	1.451 (0.564–3.731)	0.436	1.466 (0.551–3.900)	0.440
Walking	84 (45.9)	32 (42.0)	0.855 (0.478–1.527)	0.593	0.627 (0.311–1.266)	0.191

n = unweighted number of study population. Multivariate analysis was adjusted for age, sex, residence, household number, education, marriage, asthma, atopic dermatitis, and stress.

**Table 5:** Odds ratios for the associations between physical activity and (A) total, (B) *D. farinae*, (C) dog, and (D) cockroach IgE levels (log 10 transformed) in patients with allergic rhinitis.

**A**						
	**Mean ± SE**		**Univariate**		**Multivariate**	
	**No PA**	**PA**	**Odds Ratio** **(95% CI)**	***p***	**Odds Ratio** **(95% CI)**	***p***
Vigorous	2.33 ± 0.047	2.24 ± 0.119	0.750 (0.337–1.671)	0.479	0.420 (0.161–1.095)	0.076
Moderate	2.30 ± 0.046	2.40 ± 0.126	1.343 (0.600–3.005)	0.470	1.531 (0.531–4.415)	0.427
Walking	2.32 ±0.068	2.30 ± 0.065	0.945 (0.510–1.750)	0.856	0.771 (0.381–1.560)	0.466
**B**						
	**Mean ± SE**		**Univariate**		**Multivariate**	
	**No PA**	**PA**	**Odds Ratio** **(95% CI)**	***p***	**Odds Ratio** **(95% CI)**	***p***
Vigorous	0.51 ± 0.081	0.63 ± 0.136	1.150 (0.808–1.636)	0.435	1.066 (0.690–1.646)	0.773
Moderate	0.54 ± 0.079	0.49 ± 0.182	0.947 (0.605–1.483)	0.811	0.887 (0.553–1.421)	0.615
Walking	0.52 ± 0.091	0.55 ± 0.125	1.032 (0.718–1.482)	0.865	0.895 (0.602–1.328)	0.578
**C**						
	**Mean ± SE**		**Univariate**		**Multivariate**	
	**No PA**	**PA**	**Odds Ratio** **(95% CI)**	***p***	**Odds Ratio** **(95% CI)**	***p***
Vigorous	−1.06 ± 0.057	−1.04 ± 0.132	1.032 (0.600–1.774)	0.910	0.793 (0.396–1.587)	0.509
Moderate	−1.07 ± 0.058	−0.97 ± 0.129	1.202 (0.720–2.006)	0.479	1.391 (0.728–2.659)	0.315
Walking	−1.06 ± 0.081	−1.05 ± 0.083	1.026 (0.635–1.659)	0.915	0.902 (0.559–1.455)	0.669
**D**						
	**Mean ± SE**		**Univariate**		**Multivariate**	
	**No PA**	**PA**	**Odds Ratio** **(95% CI)**	***p***	**Odds Ratio** **(95% CI)**	***p***
Vigorous	−0.60 ± 0.060	−0.71 ± 0.143	0.803 (0.423–1.522)	0.498	0.550 (0.271–1.116)	0.097
Moderate	−0.62 ± 0.056	−0.64 ± 0.162	0.961 (0.501–1.846)	0.905	0.932 (0.452–1.919)	0.846
Walking	−0.60 ± 0.075	−0.64 ± 0.069	0.920 (0.633–1.337)	0.660	0.880 (0.577–1.341)	0.549

PA: physical activity.

## Supplementary Materials

The following are available online at https://www.mdpi.com/1660-4601/17/16/5662/s1, Table S1: Odds ratios for the associations between physical activity and severity or persistence of rhinitis of non-allergic rhinitis. Table S2: Frequency analysis of allergen-specific IgE levels divided into six grades.
