# Supplementary material for: Association between Allergic Rhinitis and Regular Physical Activity in Adults: A Nationwide Cross-Sectional Study
Source: Int J Environ Res Public Health. 2020 Aug 5;17(16):5662. doi: 10.3390/ijerph17165662 (PMC7459676; doi:10.3390/ijerph17165662)
Supplement: Supplementary file 1 [file ijerph-17-05662-s001.pdf]

Supplementary

Table S1. Odds ratios for the association between physical activity and severity or persistence of non-allergic rhinitis

Severity

	n (%)		Univariate		Multivariate	
	Mild<br>n =175	Moderate-severe<br>n = 49	Odds ratio (95% CI)	*P*	Odds ratio (95% CI)	*P*
Vigorous	26 (12.7)	9 (18.5)	1.558 (0.620-3.916)	0.342	1.588 (0.547-4.611)	0.391
Moderate	17 (11.6)	3 (3.9)	0.306 (0.114-0.821)	0.019	0.267 (0.088-0.812)	0.021
Walking	79 (39.0)	18 (45.2)	1.289 (0.594-2.798)	0.516	1.161 (0.559-2.412)	0.686

Persistence

	n (%)		Univariate		Multivariate	
	Intermittent<br>n = 177	Persistent<br>n = 47	Odds ratio (95% CI)	*P*	Odds ratio (95% CI)	*P*
Vigorous	30 (15.6)	5 (6.8)	0.393 (0.104-1.476)	0.164	0.357 (0.085-1.503)	0.158
Moderate	18 (11.1)	2 (5.5)	0.467 (0.083-2.627)	0.384	0.519 (0.093-2.891)	0.451
Walking	78 (39.9)	19 (41.9)	1.083 (0.494-2.373)	0.840	0.940 (0.411-2.151)	0.882

n = unweighted number of study population.

Table S2. Frequency analysis of allergen-specific IgE levels divided into six grades

Dermatophagoides farinae

	n (%)							
	Total	I	II	III	IV	V	VI	*P*
Vigorous								
Yes	47 (100)	6 (5.2)	16 (31.5)	13 (35.8)	6 (16.6)	2 (4.2)	4 (6.8)	0.453
No	194 (100)	26 (13.5)	54 (25.9)	48 (29.3)	29 (12.8)	26 (12.3)	11 (6.3)	
Moderate								
Yes	28 (100)	3 (13.0)	7 (21.9)	10 (40.5)	3 (11.1)	3 (9.3)	2 (4.2)	0.926
No	213 (100)	29 (11.7)	63 (27.7)	51 (29.3)	32 (13.9)	25 (10.8)	13 (6.6)	
Walking								
Yes	107 (100)	12 (11.0)	35 (26.5)	24 (31.7)	16 (14.3)	11 (7.9)	9 (8.6)	0.787
No	134 (100)	20 (12.7)	35 (27.4)	37 (29.7)	19 (12.9)	17 (12.9)	6 (4.4)	

Dog

	n (%)							
	Total	I	II	III	IV	V	VI	*P*
Vigorous								
Yes	7 (100)	2 (22.6)	3 (43.9)	2 (33.5)	0 (0)	0 (0)	0 (0)	0.717
No	37 (100)	14 (41.9)	15 (35.4)	7 (22.2)	1 (0.5)	0 (0)	0 (0)	
Moderate								
Yes	7 (100)	3 (39.5)	3 (46.2)	1 (14.2)	0 (0)	0 (0)	0 (0)	0.868
No	37 (100)	13 (38.8)	15 (35.5)	8 (25.2)	1 (0.5)	0 (0)	0 (0)	
Walking								
Yes	19 (100)	5 (21.6)	10 (52.4)	4 (25.9)	0 (0)	0 (0)	0 (0)	0.255
No	25 (100)	11 (49.9)	8 (26.7)	5 (22.7)	1 (0.7)	0 (0)	0 (0)	

Cockroach

	n (%)							
	Total	I	II	III	IV	V	VI	*P*
Vigorous								
Yes	17 (100)	8 (52.0)	7 (34.2)	2 (13.8)	0 (0)	0 (0)	0 (0)	0.899
No	89 (100)	33 (44.7)	40 (39.9)	13 (13.6)	3 (1.8)	0 (0)	0 (0)	
Moderate								
Yes	13 (100)	6 (49.9)	5 (29.9)	2 (20.2)	0 (0)	0 (0)	0 (0)	0.835
No	93 (100)	35 (45.4)	42 (40.0)	13 (12.9)	3 (1.7)	0 (0)	0 (0)	
Walking								
Yes	45 (100)	15 (38.5)	22 (46.3)	7 (14.0)	1 (1.3)	0 (0)	0 (0)	0.654
No	61 (100)	26 (51.2)	25 (33.7)	8 (13.4)	2 (1.8)	0 (0)	0 (0)	

n = unweighted number of study population. IgE levels were divided into 6 grades: Grade I (0.35–0.69 kU/L), Grade II (0.70–3.49 kU/L), Grade III (3.50–17.49 kU/L), Grade IV (17.50–49.99 kU/L), Grade V (50.00–100.00 kU/L), and Grade VI (> 100.00 kU/L)
